# Understanding how interculturality is taught and learned by students, professors, and academic authorities through phenomenography

**DOI:** 10.1590/1980-220X-REEUSP-2025-0428en

**Published:** 2026-05-25

**Authors:** Claudia Verónica Pérez Acuña, José Luis Medina

**Affiliations:** 1 Clínica Alemana Universidad del Desarrollo, Facultad de Medicina, Santiago, Chile.; 2 Clínica Alemana Universidad del Desarrollo, Facultad de Medicina, Instituto de Ciencia e Innovación en Medicina, Centro de Salud Global Intercultural, Santiago, Chile.; 3 Universidad de Barcelona, Facultad de Educación, Barcelona, España.

**Keywords:** Cultural Competency, Learning, Health Personnel, Competência Cultural, Aprendizagem, Pessoal de Saúde

## Abstract

**Objective:**

To understand how interculturality is taught and learned by how interculturality in the field of health is taught and learned from the perspectives of students, professors, and academic authorities involved in health-related programs within a faculty of medicine.

**Method:**

This research employs phenomenographic qualitative methods that include semi-structured interviews with 27 key agents, including students, faculty members, and program and academic authorities. The data analysis process involved the seven stages of phenomenographic analysis.

**Results:**

Two approaches were identified: (A) Theoretical-experiential approach: academic authorities and professors highlight the importance of combining theory with experiential strategies, such as workshops, simulations, and international collaborative learning; (B) Direct experience with clinical practice: immersion in real contexts is emphasized on the basis of clinical rotations, tutor modelling, and participation in migrant clinics. Students highlight the value of volunteering and academic exchange.

**Conclusion:**

Training healthcare professionals in interculturality requires combining theory, practice, experiential activities, and assessment, supported by strategies fostering knowledge, attitudes, and skills for inclusive, culturally competent healthcare in increasingly diverse contexts.

## RESUMO

**Objetivo:**

Compreender como a interculturalidade é ensinada e aprendida na área da saúde, a partir das perspectivas de estudantes, professores e autoridades acadêmicas envolvidas em programas relacionados à saúde em uma faculdade de medicina.

**Método:**

Esta pesquisa emprega métodos qualitativos fenomenográficos, incluindo entrevistas semiestruturadas com 27 agentes-chave, entre estudantes, docentes e autoridades acadêmicas e de programas. O processo de análise de dados envolveu as sete etapas da análise fenomenográfica.

**Resultados:**

Foram identificadas duas abordagens: (A) Abordagem teórico-experiencial: autoridades acadêmicas e professores destacam a importância de combinar a teoria com estratégias experienciais, como workshops, simulações e aprendizagem colaborativa internacional; (B) Experiência direta com a prática clínica: a imersão em contextos reais é enfatizada com base em estágios clínicos, modelagem por tutores e participação em clínicas para migrantes. Os alunos destacam o valor do voluntariado e do intercâmbio acadêmico.

**Conclusão:**

A formação de profissionais de saúde em interculturalidade exige a combinação de teoria, prática, atividades vivenciais e avaliação, apoiada por estratégias que promovam conhecimento, atitudes e habilidades para uma assistência à saúde inclusiva e culturalmente competente em contextos cada vez mais diversos.

## INTRODUCTION

Globalization and cultural diversity have highlighted the importance of interculturality in the field of health^(1)^. Before addressing the challenges that result from interculturality within the health sector, it is essential to define culture as a “complex and dynamic set of beliefs, knowledge, values and behaviors that are learned and transmitted through language and life in society”^(2,8)^. In this context, interculturality is not limited to the coexistence of diverse cultures but rather implies an active process of exchange, in which differences are accepted and valued in the context of healthcare^(3)^. Intercultural competency (IC) is defined as “the ability to generate meaningful human encounters in various health settings, in which experiences, emotions related to discomfort, world views, identity, beliefs, relationships with the health system, and corporeality and disease are shared in a manner that depends on each person and their role in the interaction”^(3,10)^.

This situation presents a significant challenge in training healthcare professionals to provide quality, equitable care, who must be able to provide quality and equitable care, in which context all people must be offered the opportunity to receive the same care regardless of their race, ethnicity, sex, gender identity, sexual orientation, disability, training, employment, religion, language, address, among other factors^(1,4)^. Cultivating a culturally competent healthcare service has been identified as a key factor in improving health outcomes, increasing clinical staff efficiency, and promoting patient satisfaction^(5)^.

The World Health Organization has identified IC as an essential component of health education^(6)^. A study on this topic reported that professionals with high intelligence quotients provide more appropriate and personalized care, experience higher levels of job satisfaction and exhibit a better understanding of the needs of their patients^(7)^. These considerations must be integrated effectively into the curricula used in relevant training programs^(8)^. Some strategies and tools that have been used to teach interculturality include cooperative learning, improvisation, research-based instruction, task-based instruction, production, interaction, negotiation, mediation, and roleplaying exercises^(9)^.

In the context of health-related programs, this topic has gained increasing interest, particularly in medicine, where traditional approaches have been criticized for placing excessive emphasis on biomedicine and for failing to adequately consider the cultural and social contexts that influence patients’ health and well-being^(10)^. In response to this criticism, numerous educational institutions have begun to integrate interculturality into their curricula with the aim of preparing students to work in culturally diverse clinical settings.

The incorporation of interculturality into the curricula of health-related programs, however, involves significant challenges. Recent studies have highlighted the importance of implementing diverse pedagogical strategies, such as simulations, group discussions, case studies, and critical reflections, to promote the development of ICs. These strategies not only enrich theoretical learning but also prepare students to address real situations^(11,12,13)^.

In Chile, as in other countries worldwide, healthcare professionals must be prepared to provide care to an increasingly heterogeneous population. Although interculturality has been integrated into the curricula used in the fields of medicine and nursing, its implementation in other health disciplines, such as dentistry, physiotherapy, speech therapy, and medical technology, remains at an early stage^(14,15)^.

In this context, this study was designed to address a central question: in what qualitatively different ways do students, faculty members, and academic authorities in a school of medicine understand how interculturality is taught and learned in healthcare professionals’ education? Therefore, this study aims to understand how interculturality in the field of health is taught and learned from the perspectives of students, faculty members, and academic authorities involved in health-related programs within a school of medicine affiliated with a Chilean university.

## METHOD

### Study Design

This is an exploratory and qualitative study that is rooted in a phenomenographic approach. Phenomenography aimed to study the variations in people’s conceptions of or ways of understanding a given phenomenon in the surrounding world^(16)^. This approach has begun to be used in the field of healthcare in the past 20 years^(17)^. The focus of phenomenography is on the set of qualitatively different ways in which a given phenomenon can be experienced^(16)^. It is based on the assumption that any phenomenon can be understood in a limited number of ways^(17)^. Since the focus of phenomenography lies in identifying the set of qualitatively different ways of experiencing a phenomenon^(18)^, this approach was selected due to the interest in qualitatively mapping the full range of possible experiences of the individuals who participated in the study.

### Local

The study was conducted at a private university in Santiago de Chile, in the School of Medicine.

### Population and Selection Criteria

A purposive sampling strategy was used to include students and faculty members. In other words, individuals who were willing to participate in the study were selected after being contacted through the university’s official communication channels. To maximize variation in participants’ qualitative experiences regarding the phenomenon under study, faculty and program authorities were invited to participate in order to ensure the inclusion of participants with diverse onto-epistemological perspectives.

A purposive sampling strategy was employed for participant selection. To ensure sufficient variability, three principles were followed: (a) each student, faculty member, and authority had some degree of intercultural experience (e.g., participation in an exchange semester, coming from a region other than the Metropolitan Region, belonging to an Indigenous group, among others); however, this level was not necessarily equivalent, which was considered to ensure the greatest possible variation in their experiences. (b) Participants differed in terms of discipline, university role, age, professional or academic experience, and gender (including men and women). Finally, (c) the sample consisted of 27 participants: (i) students from medicine, medical technology, nursing, nutrition, physical therapy, speech-language pathology, and dentistry (n = 11); (ii) faculty members (n = 5); and (iii) program authorities and healthcare professionals (n = 11). To maintain sufficient diversity, as recommended in phenomenography, the suggested sample size ranges between 15 and 30 participants. Participant characteristics are presented in Table 1.

**Table 1:** Participant characteristics – Santiago, Chile, 2022.

Code	Participants/key agent	Profession	Years of professional training/student	Years in office	Age (years)	Gender
AUT	Dean	Physician	30	7	58	Male
AUT	Vice dean	Physician	36	11	64	Female
AUT	Vice dean	Nurse	29	7	52	Female
AUT	Academic coordinator	Nurse	23	14	45	Female
AUT	Academic coordinator	Physiotherapist	25	19	47	Male
AUT	Program director	Nutritionist	29	15	52	Female
AUT	Program director	Physiotherapist	33	15	56	Male
AUT	Program director	Medical technology	42	15	66	Female
AUT	Program director	Speech therapist	39	15	62	Female
AUT	Program director	Physician	21	5	46	Female
AUT	Program director	Dentist	30	6	54	Male
TEA-NUT	Faculty member	Nutritionist	20	8	44	Female
TEA-PHY	Faculty member	Physiotherapist	17	5	40	Male
TEA-NUR	Faculty member	Nurse	22	10	46	Female
TEA-NUR	Faculty member	Nurse	6	5	36	Female
TEA-MED	Faculty member	Physician	32	7	56	Female
MED-STU	Course student	Medicine	7	–	25	Male
MED-STU	Course student	Medicine	7	–	25	Female
DEN-STU	Course student	Dentist	5	–	23	Male
DEN-STU	Course student	Dentist	6	–	24	Male
MT-STU	Course student	Medical technology	5	–	23	Male
NUR-STU	Course student	Nurse	5	–	22	Female
NUR-STU	Course student	Nurse	5	–	24	Female
NUT-STU	Course student	Nutritionist	5	–	22	Female
PHY-STU	Course student	Physiotherapist	5	–	23	Male
ST-STU	Course student	Speech therapist	5	–	24	Female
ST-STU	Course student	Speech therapist	5	–	23	Male

Legend: AUT – STU – Medical Authority; TEA-NUT – Teaching Nutrition; TEA-PHY – Teaching Physiotherapy; TEA-NUR – Teaching Nursing; TEA-MED Student; DEN-STU – Dentistry Student; MT-STU – Medical Technology Student; NUR-STU – Nursing Student; NUT-STU – – Teaching Medicine; MEDNutrition Student; PHY-STU –

### Data Collection

Data was collected for this research between June 2020 and March 2022 by the study’s lead researcher, with experience in interculturality and in interviews for qualitative research. On the basis of semi-structured individual interviews, these interviews were conducted online via Zoom platform (https://zoom.us/). This information gathering format was chosen in response to the context of a pandemic. Only one interview was conducted with each study participant. Data were collected through a semistructured interview, which is considered one of the dominant techniques in phenomenographic research. Previous studies suggest that interviews offer the greatest potential for collecting and clarifying data in phenomenographic research^(19,20)^. To collect information, a semi-structured individual interview guide was designed. The interviews focused on two interrelated domains: (A) The first domain focused on the process of teaching interculturality in the context of training healthcare professionals, particularly in terms of how pedagogical strategies, content, and professor preparation could be used to transmit concepts, skills, and values pertaining to interculturality. It focuses on pedagogical strategies and content, as well as the preparation of professors to convey relevant knowledge and corresponding sensitivities; (B) The second domain focused on the process of learning interculturality in the context of training healthcare professionals, particularly with respect to the ways in which students acquire, internalize and apply relevant knowledge, skills, and values. In this context, we must consider their learning experiences, the resources used in this process, and the ways in which this learning can be integrated into professional training and clinical practice.

Each session lasted between 30 and 60 minutes and was conducted individually. Each interview continued until the interviewer and participant agreed that they had discussed all aspects of the phenomenon under investigation. Each interview was recorded and transcribed for subsequent analysis.

Various strategies were implemented to minimize potential biases in data collection and strengthen the credibility and transparency of the process. First, feedback was sought from colleagues and external experts to identify biased interpretations that the principal researcher might have overlooked. Additionally, a reflective research journal was maintained to record thoughts, assumptions, and analytical decisions, thereby fostering self-reflection and awareness of the researcher’s influence on the study. Finally, the researcher explicitly acknowledged their positionality and any potential influences derived from their professional and personal background. They openly communicated these aspects to participants to enhance the study’s integrity and trustworthiness.

The recordings were transcribed verbatim and verified to ensure data fidelity. The audio files were then permanently deleted once transcription and validation were complete. The anonymized transcripts and informed consent forms were stored on a password-protected institutional server accessible only to authorized research team members. In accordance with institutional policies and international recommendations on research integrity and personal data protection, the data will be retained for a period of five years.

### Date Analysis and Processing

The data analysis process involved the seven stages of phenomenographic analysis described by Dahlgren and Fallsberg^(19,20)^: familiarization with the data, condensation, comparison, grouping, articulation, labelling, and contrast. Initially, this process was performed manually, although Atlas.Ti software version 9 was subsequently used to support this analysis. To ensure the rigor of this research, multiple measures were used, including identifying the researchers’ preconceptions about the phenomenon under investigation, ensuring genuine expression of the phenomenon with the help of facilitators, reaching a consensus through negotiation, and using the following primary criteria, as described by Marton and Booth, to judge the quality of the identified categories^(20)^: 1) each category reveals a different aspect of the manner in which the phenomenon is experienced; 2) categories are characterized by logical and clear relationships with each other; and 3) final results are parsimonious (i.e., the fewest possible categories are employed).

Multiple measures were used to ensure the rigor and quality of the research. The iterative nature of the study enabled the maintenance of credibility, consistency, transferability, and reliability^(20)^. First, credibility was based on demonstrating similarities and differences supported by transcript data, with interview excerpts used to support the categories. Second, consistency was established by maintaining separate and exclusive categories and by establishing the correspondence between the results and related studies. Third, transferability and reliability were developed through the use of appropriate methodological procedures to obtain consistent, high-quality data for analysis.

This involved a meticulous design of the interview questions and documentation of each stage of the research process.

### Ethical Aspects

The study was approved by the Research Ethics Committee of the Faculty of Medicine at *Universidad del Desarrollo* (#2019-091) and the Bioethics Committee of *Universidad de Barcelona* (IRB00003099). The study adhered to the principles of voluntariness, confidentiality, and anonymity of participants, as reflected in the signing of an informed consent.

## RESULTS

The results of the analysis of the differences in the perceptions of key agents have been assigned to of two categories:

### A – Experiential Theoretical Approach

Academic authorities identified intercultural literacy as a fundamental pillar in the training of healthcare professionals.

*I see it as an intercultural literacy ...... I am sure that there will be ways to address it eventually—not only in a year but in several years—as an important issue.* (I1 AUT)

Academic authorities also emphasized the facts that the initial theoretical approach should be continuous and that it should not be limited to a single course but rather integrated throughout the entire course of academic training, thus addressing this component in a systematic and progressive manner.

*I think that I would do it progressively in the curriculum, first by raising awareness in a theoretical way in the early years and then by inserting it in some way.* (I9 AUT)

From academic authorities’ perspective, various teachinglearning strategies. These strategies included both lectures and applied research. Furthermore, novel-based methodological strategies can be used to teach narrative medicine.

... *in terms of novels, it occurs to me ... in which one lives the experience of the protagonist in that culture, including what happens. That could be an interesting way of teaching students*. (I4 AUT)

Among the variety of outstanding teaching-learning strategies that were highlighted by academic authorities, portfolios were a notable example.

*Make it a competition that is worked on from day one when they enter, asking the students themselves to identify their own culture, their family, their ancestors, even asking, I do not know, where is your name from, doing daily exercises, exposing them to cases, situations, newspapers, everyday focusing on something related to interculturality*. (I3 AUT)

For their part, faculty members indicated that interculturality in the education of healthcare professionals is primarily recognized as an implicit component of the curriculum, through various teaching–learning activities. In this context, they identified nursing as the only program that explicitly incorporates this topic into classroom content. Unlike academic authorities, faculty members acknowledged the relevance of a theoretical–experiential approach in its teaching; however, they expressed differing views regarding its implementation and the degree of emphasis it should receive within the curriculum.

Although both professors and academic authorities valued the transmission of theoretical content pertaining to the fundamental concepts of interculturality, professors emphasized the importance of the practical application of such content. They claimed that strategies such as workshops, group work, debates and simulations involving standardized patients are essential to efforts to reinforce learning and cultivate ICs effectively.

*Always start with concepts, ideas, books, and articles; always begin with something theoretical, but that theoretical component is complemented by practical elements or tools, such as workshops—workshops like those led by Andrés, which I believe are extremely necessary*. (I12 TEA-NUR)

*Participation, or the approach, teamwork, and role-playing as well. The use of imagery, working with people from different cultures. It should be something that leaves a lasting impression, like when you go to a class and a patient comes in, or when you invite the patient’s family; students never forget it.* (I14 TEA-ST)

*Develop intercultural simulation scenarios and, perhaps, incorporate high-fidelity scenarios in which the student must engage with a user, a person—for instance, someone who does not speak Spanish—I am thinking, for instance, of a Mapuche individual or a Haitian individual—and enter a simulation scenario in which they must apply what they have learned.* (I12 TEA-NUR)

Another teaching strategy that medicine and nutrition professors viewed as fundamental pertains to the multicultural classroom, which involves the participation of students and professors of different cultures (e.g., those from different countries or with different genders, ethnicities, or ages), which can promote the use of teaching strategies such as discussion and debate alongside exposure to cultural diversity in terms of opinions and perspectives.

*I think that it would also be wonderful to be able to learn from professors from other cultures and alongside students from different cultures.* (I16 TEA-MED)

Nursing and physical therapy professors tended to focus more on the direct application of this knowledge to experiential teaching situations, and they highlighted the importance of specific clinical cases with regard to effective and meaningful learning.

*We generally work a lot with clinical cases where we put ourselves in the situation of caring for a patient, and they apply the intercultural approach to the case in terms of how they would approach it and what questions they would ask*. (I13 TEA-NUR)

*The incorporation of clinical cases. We work a lot on that*. (I15 TEA-PHY)

In terms of teaching strategies, nursing professors also highlighted the incorporation of cultural facilitators. These individuals, who are also known as translators, linguistic facilitators and/or cultural mediators, are dedicated to the task of facilitating communication between the health team and the migrant population or native populations. They participate in clinical activities at different health centers and are invited to participate in theoretical activities with students.

*I also believe it could be important to address the role of the intercultural facilitator as an integral member of the healthcare team; I think this would be highly relevant.* (I13 TEA-NUR)

Similarly, faculty members emphasized that interculturality should be understood as an integral component of professionalism in healthcare, and that its teaching should be aligned with fundamental ethical and professional values. In this regard, it is essential to address and correct prejudices through activities such as cultural awareness workshops or visits to a *ruca* with a Mapuche *machi* (an intermediary between worlds). These individuals are connected to the supernatural realm and their protective spirits, which grant them powers to combat malevolent spirits; they safeguard the well-being of their patients and the community, serving as healers, particularly in the case of women. Likewise, students may participate in role-playing exercises that simulate intercultural situations.

*Yes, eliminating prejudice, I think. Because I was thinking about our first visit to a Mapuche ruca... the class was composed almost entirely of women. The machi came out and had the men from the class enter first, and then the women. Because Mapuche culture is patriarchal, and from that point the female students became upset—they did not think it was appropriate... We need to eliminate the prejudice that my way of thinking is the only valid one.* (I12 TEA-NUR)

From students’ perspective, other teaching-learning strategies were identified that different from those highlighted by other key agents, such as key cultural informants, such as a member of an indigenous population (Mapuche) or a person of a certain gender identity (transgender) who participates in workshops alongside students.

*Being able to bring people to bear witness to us is a key tool for getting to know different groups of people. Additionally, the experiences of our professors are key tools and factors that can help us understand and learn about this topic greatly*. (I22 NUR-STU)

Students also mentioned courses or activities based on the collaborative online international learning (COIL) approach to teaching, which represents an innovative strategy for teaching interculturality to health students.

*Or, for instance, share cer.tain courses with people from other universities, from other countries, as some course platforms usually do*. (I19 MT-STU)

### B – Direct Experience in Clinical Practice

In this study, academic authorities emphasized the relevance of the integration of practical experience into the process of teaching interculturality. The integration of theory and practice should not be treated in isolation but rather included cohesively and continuously in the curriculum and viewed as an essential component of an educational strategy. In particular, the importance of clinical rotations in culturally varied communities at the primary and hospital levels was highlighted, in which context students interact directly with patients from different cultures in real situations. This practical immersion helps students develop skills such as empathy and adaptability, thus ensuring that future professionals are able to apply intercultural knowledge in their daily practice.

*In the end, everything is learned through practice—by listening and observing. You can read millions of articles and books, but it will never be the same as what you experience, such as when you go to a clinical setting where people from different cultures are treated; that is a powerful form of learning*. (I6 AUT)

*I believe that students must be exposed to different clinical experiential situations—with patients, with people—in different settings*. (I3 AUT)

Academic authorities also identified modelling by clinical tutors as a teaching strategy that is fundamental with respect to the process of teaching interculturality to health students. The interaction between tutors and students within a clinical context allows future professionals to observe and learn how to apply intercultural skills in real situations, and tutors serve as experiential examples in this context.

*I think the concepts can be taught in the classroom during the early years. However, they should later be developed through modeling. At that point, the tutor must be immersed in this process in order to effectively convey it to students*. (I10 AUT)

... *modelling; students do not do what you say, students do what you do. I don’t know; in my case, for instance, I have never forgotten those professors who taught me well, and I have never forgotten those professors who taught me poorly*. (I2 AUT)

Direct experience in the clinical field was viewed as essential with respect to the process of learning interculturality by both professors and academic authorities. However, the approaches they mentioned varied in terms of how such experiences were to be. They emphasize the importance of preparing students adequately before their exposure to diverse clinical settings as well as that of accompanying them during these experiences with the goal of ensuring that they could acquire the necessary competencies.

*I would try to ensure that all my students have an experience with a patient from another country, with a different belief system, or from a different ethnicity or race. For instance, I would try to ensure that they do not feel that fear when facing someone from another culture in the future.* (I16 TEA-MED)

A teaching strategy that was highlighted by nursing professors pertained to the migrant clinic, which is a key practical activity that can facilitate the development of ICs among health students. These practical experiences provide a unique opportunity for students to confront the reality of serving vulnerable and culturally diverse populations.

*I believe that one of these ways is through the migrant clinic, where the student has the opportunity to interact with patients who are just entering the health system in a very good way.* (I13 TEA-NUR)

Professors emphasized the fact that interculturality assessment should not be limited to theoretical exams but should also include tools that can help measure the practical performance of students in real settings. Professors mentioned the importance of assessing students’ ability to adapt to intercultural situations as well as their competency in interacting with patients of diverse cultural origins.

*I think that in more advanced courses, for instance, in the third, fourth, and fifth years, the assessment criteria— especially during clinical rotations—should include an explicit way to assess whether the student is able to integrate interculturality into their healthcare practice*. (I12 TEA-NUR)

From students’ perspective, in addition to the strategies mentioned by academic authorities and faculty members, non-mandatory practical experiences were identified, such as volunteer programs at both the local and national levels. These opportunities allow direct interaction with diverse populations, facilitating the development of intercultural competencies in real-world contexts.

... *I can think of examples of this occurring in programs such as Operation Smile, Calle Salud, and similar initiatives...* (I17 MED-STU)

... *the university also gives us the opportunity to engage in volunteer work, and I think it is one of the best ways to get to know the different cultures that exist not only at the city level, such as in Santiago, but also at the national level*. (I23NUR-STU)

Students also mentioned international academic exchange programs, which they identified as a highly effective pedagogical strategy that can be used to cultivate ICs among health students.

*The exchanges, as they come and go*. (I18 MED-STU)

... *there are different exchanges for various places, and they are also very open to the possibility of exchange students arriving here*. (I22 NUR-STU)

In summary, two approaches are identified: (A) Theoreticalexperiential approach: experts and faculty emphasize the importance of combining theory with experiential strategies, such as workshops, simulations, and international collaborative learning; (B) Direct experience in clinical practice: emphasis is placed on immersion in real-world contexts through clinical rotations, mentoring, and participation in clinics for migrants. Students highlight the value of volunteering and academic exchange.

## DISCUSSION

Our study has several limitations, which pertain mainly to possible sources of bias and the limited generalizability of these findings. These limitations include potential recall bias or subjective responses. Furthermore, the design of this research, which involved phenomenographic methodology, focuses on the variations among experiences rather than on generalization.

The processes of teaching and learning interculturality in the context of training healthcare professionals are characterized by notable differences in the perceptions and approaches that are highlighted by academic authorities, professors, and students. This research revealed that training in interculturality among healthcare professionals is a complex process, which must take into account both the acquisition of theoretical knowledge and the practical application of intercultural skills in health education, thus enabling future healthcare professionals to feel that they are prepared to provide care or enabling them to focus on patients in an intercultural context, since previous studies have suggested that a lack of preparation persists among healthcare professionals^(21,22)^.

The findings of this study regarding academic authorities underscore the necessity of intercultural literacy that is continuous and transversal. Such a focus on literacy must be integrated throughout the academic curriculum, which represents a significant challenge to institutional structures in efforts to incorporate this content into all health programs’ curricula. This approach allows relevant stakeholders to ensure students internalize these principles from the beginning of their training. This approach aligns with research that has emphasized the importance of explicitly and systematically incorporating interculturality into the curriculum from the outset of the training process^(4)^. This approach entails not only the inclusion of theoretical content pertaining to cultural diversity but also the promotion of critical reflection on one’s own prejudices and biases^(12)^. This reflection can be facilitated by an analysis of lived experiences, thus helping students organize and communicate their thoughts and enabling them to determine whether they truly understand a given topic^(23,24)^. In this sense, professors believe that this approach must be supported by diverse pedagogical strategies, such as case studies and reflective portfolios. These strategies have been reported to effectively sensitize students to the importance of culturally competent healthcare.

Regarding the theoretical-experiential approach, the professors in this study emphasized that interculturality cannot be examined solely from a theoretical standpoint. Rather, it must be accompanied by experiential teaching and learning strategies that enable students to apply their knowledge to real-life situations in the classroom. This encourages introspection, the exchange of experiences, collaborative learning, and awareness of cultural diversity^(12,13,22)^.

In addition to the traditional strategies identified by this study, such as problem-based learning, case study analysis, and simulations involving standardized patients, the inclusion of patients from different genders or with different beliefs can provide a controlled environment in which students can improve their skills and confidence. Other possibilities pertain to participation in workshops involving cultural informants and direct contact with diverse populations. For instance, interacting with indigenous peoples, such as the Mapuche, or people with diverse gender identities, can give students a direct understanding of the barriers these populations face in the health system and their needs^(8,12,22)^. The literature has reported that this type of intervention can facilitate the development of essential skills pertaining to the care of diverse populations and promote greater sensitivity towards the sociocultural determinants of health^(3,13)^. Furthermore, research on intergroup contact has suggested that exposure to groups that have historically faced prejudice can transform negative attitudes, thereby promoting a more understanding and empathetic perspective among future healthcare professionals^(23)^.

The professors included in this study also indicated that intercultural classrooms and professors can be used in this context. Intercultural contact between professors of different backgrounds and students offers valuable opportunities for students to develop IC^(10)^. First, the diversity of opinions and perspectives can be highlighted in group discussions, which can be enriched by the group’s heterogeneity. Second, students observe how cultural behaviors that they have previously learned theoretically can be applied to everyday situations within the classroom. Third, interaction in multicultural rooms allows students to acquire knowledge regarding different cultures directly from their professors and peers, thus enriching their understanding^(13)^.

Another new emerging methodology identified by students is the COIL approach, which facilitates interaction among students from different countries and cultures, thus enriching intercultural learning without the need for physical mobility^(24)^. According to recent studies, the implementation of the COIL approach in the context of health education is an effective tool that can be used to increase IC^(25)^.

Direct experience in clinical practice is also a fundamental aspect of intercultural education^(22)^, and our data support the link between clinical experiences and preparation for intercultural care. Direct interaction with migrant patients, people of different genders, and members of other cultural groups, which often entails difficulties in terms of language, social, and cultural factors, allows students to effectively apply their theoretical knowledge of interculturality. In this context, a teachinglearning strategy involves rotations or clinical practice in diverse communities. This provides students with invaluable opportunities to apply their knowledge in real situations, an approach that has received strong support from the literature^(22,26)^.

Modelling by clinical professors (tutors) is fundamental with respect to teaching interculturality in the context of training healthcare professionals. These tutors not only serve as references with regard to the application of IC pertaining to daily clinical practice but also reinforce students’ theoretical learning, thus strengthening their confidence in their ability to manage culturally diverse clinical settings. The literature has supported the claim that effective modelling contributes significantly to the development of empathy and intercultural communication capacity among students, thereby increasing their preparation to act in clinical situations involving patients from different cultural contexts^(6,27)^. However, the literature has also highlighted multiple barriers that can hinder the integration of this training, including institutional restrictions, resistance to change by students and a lack of continuous support for educational initiatives in this context^(28)^.

Another methodology mentioned in this context was the migrant clinic. It provides a rich and challenging learning environment and promotes the development of critical skills, such as empathy, problem-solving, and cultural adaptation. These skills are key to ensuring that future healthcare professionals provide inclusive and culturally competent care^(10,22)^.

Students highlighted direct exposure to intercultural contexts through activities such as exchanges or volunteering, which have proven to be key facilitators of the development of these competencies^(29,30)^.These experiences allow students to interact directly with culturally diverse populations at both the local and international levels, thus fostering greater openness and cultural sensitivity, which are essential elements of quality healthcare in an increasingly diverse world. Learning through volunteering exposes students to different cultural contexts, thus improving not only their understanding of the particularities of each group but also their ability to adapt their practices to the specific cultural needs of the people whom they serve^(29)^. Participation in these programmers is an effective pedagogical tool that should be integrated more widely into health education curricula^(30)^.

Finally, our results underscore the importance of assessing IC, particularly in clinical settings. According to the literature, interculturality assessment should extend beyond theoretical examinations to include observations in clinical settings, simulations with formative feedback, and analyses of reflective portfolios^(10,12,22)^.

Cultural competency assessment in health training curricula is an area that can be further developed. Designing a robust, structured assessment system that incorporates clear rubrics and both formative and summative assessment methods is essential to ensuring meaningful learning.

Future researchers could replicate this study at other universities and further explore how interculturality is taught and learned across health programs, as research on this subject remains limited.

## CONCLUSION

This study highlights the relevance of the integration of interculturality into the training in a transversal manner. A continuous approach that combines theory with practice throughout the educational process is necessary. The process of teaching interculturality must be based on a combination of experiences, collaborative teaching-learning methodologies and critical reflection tools that can offer students a deeper understanding of cultural diversity.

Modelling by clinical tutors and practical experience in culturally diverse settings, such as migrant clinics, are essential components. These experiences not only facilitate the acquisition of technical skills but also promote the development of empathy and adaptability, which are fundamental elements of an inclusive and culturally sensitive.

This research provides a novel contribution to the field by offering a comprehensive phenomenographic analysis of how interculturality is taught and learned within health education. Unlike previous studies, which have mainly focused on assessing cultural competency through isolated interventions or theoretical approaches, this study integrates the perspectives of academic authorities, professors, and students to reveal the complexity and variability of these experiences. The identification of emergent strategies—such as the inclusion of COIL, intercultural classrooms, and experiential clinical models—adds new insights into how IC can be effectively developed. These findings advance current knowledge by emphasizing that intercultural education must be approached as a continuous, multidimensional process that bridges theory, practice, and reflection throughout professional training.

## Data Availability

The entire dataset supporting the results of this study was published in the article itself.
