# Supplementary material for: Understanding how interculturality is taught and learned by students, professors, and academic authorities through phenomenography
Source: Rev Esc Enferm USP. 2026 May 25;60:e20250428. doi: 10.1590/1980-220X-REEUSP-2025-0428en (PMC13200786; doi:10.1590/1980-220X-REEUSP-2025-0428en)
Supplement: Translation

# Translation: Comprender cómo la interculturalidad es enseñada y aprendida por estudiantes, docentes y autoridades mediante la fenomenografía

**DOI:** 10.1590/1980-220X-REEUSP-2025-0428es

**Authors:** Claudia Verónica Pérez Acuña, José Luis Medina

**Affiliations:** 1 Clínica Alemana Universidad del Desarrollo, Facultad de Medicina, Santiago, Chile.; 2 Clínica Alemana Universidad del Desarrollo, Facultad de Medicina, Instituto de Ciencia e Innovación en Medicina, Centro de Salud Global Intercultural, Santiago, Chile.; 3 Universidad de Barcelona, Facultad de Educación, Barcelona, España.

**Keywords:** Competencia Cultural, Aprendizaje, Personal de Salud

## Abstract

**Objetivo:**

Comprender cómo la interculturalidad en el ámbito de la salud es enseñada y aprendida desde las perspectivas de estudiantes, docentes y autoridades vinculadas a carreras del área de la salud en una facultad de medicina.

**Método:**

Esta investigación emplea un enfoque cualitativo fenomenográfico que incluye entrevistas semiestructuradas a 27 actores clave, entre ellos estudiantes, docentes y autoridades de carrera y facultad. El proceso de análisis de datos consideró los siete pasos del análisis fenomenográfico.

**Resultados:**

Se identifican dos enfoques: (A) Enfoque teórico-experiencial: las autoridades y los docentes destacan la importancia de combinar la teoría con estrategias experienciales, tales como talleres, simulaciones y aprendizaje colaborativo internacional; (B) Experiencia directa en la práctica clínica: se enfatiza la inmersión en contextos reales a partir de rotaciones clínicas, modelamiento de tutores y participación en clínica para población migrante. Los estudiantes destacan el valor del voluntariado y del intercambio académico.

**Conclusión:**

La formación de profesionales de la salud en interculturalidad requiere combinar teoría, práctica, actividades experienciales y evaluación, apoyadas en estrategias que fomenten conocimientos, actitudes y habilidades para una atención inclusiva y culturalmente competente en contextos cada vez más diversos.

## INTRODUCCIÓN

La globalización y la diversidad cultural han puesto de relieve la importancia de la interculturalidad en el ámbito de la salud^(1)^. Antes de abordar los desafíos que plantea la inter-culturalidad en el sector de la salud, es esencial definir la cultura como un «conjunto complejo y dinámico de creencias, conocimientos, valores y comportamientos que se aprenden y transmiten a través del lenguaje y la vida en sociedad»^(2,8)^. En este contexto, la interculturalidad no se limita a la coexistencia de diversas culturas, sino que implica un proceso activo de intercambio, en el que las diferencias se aceptan y valoran en el contexto de la atención de la salud^(3)^. La competencia intercultural (CI) se define como «la capacidad de generar encuentros humanos significativos en diversos entornos de salud, en los que se comparten experiencias, emociones relacionadas con el malestar, visiones del mundo, identidad, creencias, relaciones con el sistema de salud, y corporalidad y enfermedad, de una manera que depende de cada persona y de su rol en la interacción»^(3,10)^.

Esta situación presenta un desafío significativo en la formación de los profesionales de la salud para que brinden una atención de calidad y equitativa, en cuyo contexto se debe ofrecer a todas las personas la oportunidad de recibir la misma atención, independientemente de su raza, etnia, sexo, identidad de género, orientación sexual, discapacidad, formación, empleo, religión, idioma o domicilio, entre otros factores^(1,4)^. Se ha identificado el fomento de un servicio de salud culturalmente competente como un factor clave para mejorar los resultados de salud, aumentar la eficiencia del personal clínico y promover la satisfacción del paciente^(5)^.

La Organización Mundial de la Salud ha identificado la CI como un componente esencial de la educación en salud^(6)^. Un estudio sobre este tema informó que los profesionales con altos coeficientes de inteligencia brindan una atención más adecuada y personalizada, experimentan mayores niveles de satisfacción laboral y muestran una mejor comprensión de las necesidades de sus pacientes^(7)^. Estas consideraciones deben integrarse de manera efectiva en los planes de estudio utilizados en los programas de formación pertinentes^(8)^. Algunas estrategias y herramientas que se han utilizado para enseñar la interculturalidad incluyen el aprendizaje cooperativo, la improvisación, la enseñanza basada en la investigación, la enseñanza basada en tareas, la producción, la interacción, la negociación, la mediación y los ejercicios de juego de roles^(9)^.

En el contexto de los programas relacionados con la salud, este tema ha suscitado un interés creciente, particularmente en medicina, donde los enfoques tradicionales han sido criticados por poner un énfasis excesivo en la biomedicina y por no considerar adecuadamente los contextos culturales y sociales que influyen en la salud y el bienestar de los pacientes^(10)^. En respuesta a estas críticas, numerosas instituciones educativas han comenzado a integrar la interculturalidad en sus planes de estudio con el objetivo de preparar a los estudiantes para trabajar en entornos clínicos culturalmente diversos.

Sin embargo, la incorporación de la interculturalidad en los planes de estudio de los programas relacionados con la salud implica importantes desafíos. Estudios recientes han destacado la importancia de implementar diversas estrategias pedagógicas, como simulaciones, discusiones grupales, estudios de casos y reflexiones críticas, para promover el desarrollo de las CI. Estas estrategias no solo enriquecen el aprendizaje teórico, sino que también preparan a los estudiantes para abordar situaciones reales^(11,12,13)^.

En Chile, al igual que en otros países del mundo, los profesionales de la atención de salud deben estar preparados para atender a una población cada vez más heterogénea. Aunque la interculturalidad se ha integrado en los planes de estudio utilizados en los campos de la medicina y la enfermería, su implementación en otras disciplinas de la atención de salud, como la odontología, la fisioterapia, la fonoaudiología y la tecnología médica, se encuentra aún en una etapa inicial^(14,15)^.

En este contexto, este estudio se diseñó para abordar una pregunta central: ¿de qué maneras cualitativamente diferentes entienden los estudiantes, los docentes y las autoridades académicas de una facultad de medicina cómo se enseña y se aprende la interculturalidad en la formación de los profesionales de la salud? Por lo tanto, este estudio tiene como objetivo comprender cómo se enseña y se aprende la interculturalidad en el campo de la salud desde las perspectivas de los estudiantes, los docentes y las autoridades académicas involucrados en programas relacionados con la salud dentro de una facultad de medicina afiliada a una universidad chilena.

## MÉTODO

### Diseño del Estudio

Este es un estudio exploratorio y cualitativo que se basa en un enfoque fenomenográfico. La fenomenografía tiene como objetivo estudiar las variaciones en las concepciones o formas de entender un fenómeno dado en el mundo circundante^(16)^. Este enfoque ha comenzado a utilizarse en el campo de la atención de la salud en los últimos 20 años^(17)^. La fenomenografía se centra en el conjunto de formas cualitativamente diferentes en las que se puede experimentar un fenómeno dado^(16)^. Se basa en la suposición de que cualquier fenómeno puede entenderse de un número limitado de maneras^(17)^. Dado que el enfoque de la fenomenografía radica en identificar el conjunto de formas cualitativamente diferentes de experimentar un fenómeno^(18)^, se seleccionó este enfoque debido al interés en mapear cualitativamente toda la gama de experiencias posibles de las personas que participaron en el estudio.

### Local

El estudio se llevó a cabo en una universidad privada de Santiago de Chile, en la Facultad de Medicina.

### Población y Criterios de Selección

Se utilizó una estrategia de muestreo intencional para incluir a estudiantes y miembros del cuerpo docente. En otras palabras, se seleccionó a las personas que estaban dispuestas a participar en el estudio después de haber sido contactadas a través de los canales de comunicación oficiales de la universidad. Para maximizar la variación en las experiencias cualitativas de los participantes con respecto al fenómeno en estudio, se invitó a participar al cuerpo docente y a las autoridades del programa con el fin de garantizar la inclusión de participantes con perspectivas onto-epistemológicas diversas.

Se empleó una estrategia de muestreo intencional para la selección de participantes. Para garantizar una variabilidad suficiente, se siguieron tres principios: (a) cada estudiante, miembro del cuerpo docente y autoridad tenía algún grado de experiencia intercultural (p. ej., participación en un semestre de intercambio, proceder de una región distinta a la Región Metropolitana, pertenecer a un grupo indígena, entre otros); sin embargo, este nivel no era necesariamente equivalente, lo cual se consideró para asegurar la mayor variación posible en sus experiencias. (b) Los participantes diferían en cuanto a disciplina, función universitaria, edad, experiencia profesional o académica y género (incluidos hombres y mujeres). Finalmente, (c) la muestra estuvo compuesta por 27 participantes: (i) estudiantes de medicina, tecnología médica, enfermería, nutrición, fisioterapia, fonoaudiólogo y odontología (n = 11); (ii) miembros del cuerpo docente (n = 5); y (iii) autoridades de los programas y profesionales de la salud (n = 11). Para mantener una diversidad suficiente, tal como se recomienda en la fenomenografía, el tamaño de muestra sugerido oscila entre 15 y 30 participantes. Las características de los participantes se presentan en la Tabla 1.

**Tabla 1:** Características de los participantes – Santiago, Chile, 2022

Código	Participantes/agente clave	Profesión	Años de formación profesional/estudiante	Años de trabajo	Edad (años)	Género
AUT	Decano	Médico	30	7	58	Masculino
AUT	Vicedecano	Médico	36	11	64	Femenino
AUT	Vicedecano	Enfermera	29	7	52	Femenino
AUT	Coordinador académico	Enfermera	23	14	45	Femenino
AUT	Coordinador académico	Fisioterapeuta	25	19	47	Masculino
AUT	Director del programa	Nutricionista	29	15	52	Femenino
AUT	Director del programa	Fisioterapeuta	33	15	56	Masculino
AUT	Director del programa	Tecnología médica	42	15	66	Femenino
AUT	Director del programa	Fonoaudiólogo	39	15	62	Femenino
AUT	Director del programa	Médico	21	5	46	Femenino
AUT	Director del programa	Odontólogo	30	6	54	Masculino
TEA-NUT	Miembro del cuerpo docente	Nutricionista	20	8	44	Femenino
TEA-PHY	Miembro del cuerpo docente	Fisioterapeuta	17	5	40	Masculino
TEA-NUR	Miembro del cuerpo docente	Enfermera	22	10	46	Femenino
TEA-NUR	Miembro del cuerpo docente	Enfermera	6	5	36	Femenino
TEA-MED	Miembro del cuerpo docente	Médico	32	7	56	Femenino
MED-STU	Estudiante del curso	Medicina	7	–	25	Masculino
MED-STU	Estudiante del curso	Medicina	7	–	25	Femenino
DEN-STU	Estudiante del curso	Odontólogo	5	–	23	Masculino
DEN-STU	Estudiante del curso	Odontólogo	6	–	24	Masculino
MT-STU	Estudiante del curso	Tecnología médica	5	–	23	Masculino
NUR-STU	Estudiante del curso	Enfermera	5	–	22	Femenino
NUR-STU	Estudiante del curso	Enfermera	5	–	24	Femenino
NUT-STU	Estudiante del curso	Nutricionista	5	–	22	Femenino
PHY-STU	Estudiante del curso	Fisioterapeuta	5	–	23	Masculino
ST-STU	Estudiante del curso	Fonoaudiólogo	5	–	24	Femenino
ST-STU	Estudiante del curso	Fonoaudiólogo	5	–	23	Masculino

Leyenda: AUT – Autoridad; TEA-NUT – Docencia de Nutrición; TEA-PHY – Docencia de Fisioterapia; TEA-NUR – Docencia de Enfermería; TEA-MED – Docencia de Medicina; MED-STU – Estudiante de Medicina; DEN-STU – Estudiante de Odontología; MT-STU – Estudiante de *Tecnología Médica*; NUR-STU – Estudiante de Enfermería; NUT-STU – Estudiante de Nutrición; PHY-STU – Estudiante de Fisioterapia; ST-STU – Estudiante de Fonoaudiología.

### Recolección de Datos

Los datos para esta investigación fueron recolectados entre junio de 2020 y marzo de 2022 por el investigador principal del estudio, quien cuenta con experiencia en interculturalidad y en entrevistas para la investigación cualitativa. Se trataron de entrevistas individuales semiestructuradas, realizadas en línea a través de la plataforma Zoom (https://zoom.us/). Se optó por este formato de recolección de información debido al contexto de la pandemia. Se realizó una sola entrevista con cada participante del estudio. Los datos se recopilaron mediante una entrevista semiestructurada, considerada una de las técnicas predominantes en la investigación fenomenográfica. Estudios previos sugieren que las entrevistas ofrecen el mayor potencial para recolectar y aclarar datos en la investigación fenomenográfica^(19,20)^. Para recolectar la información, se diseñó una guía de entrevista individual semiestructurada. Las entrevistas se centraron en dos ámbitos interrelacionados: (A) El primer ámbito se centró en el proceso de enseñanza de la interculturalidad en el contexto de la formación de profesionales de la salud, particularmente en términos de cómo las estrategias pedagógicas, los contenidos y la preparación de los profesores podrían utilizarse para transmitir conceptos, habilidades y valores relacionados con la interculturalidad. Se centra en las estrategias pedagógicas y los contenidos, así como en la preparación de los profesores para transmitir los conocimientos pertinentes y las sensibilidades correspondientes; (B) El segundo ámbito se centró en el proceso de aprendizaje de la interculturalidad en el contexto de la formación de profesionales de la salud, particularmente con respecto a las formas en que los estudiantes adquieren, interiorizan y aplican los conocimientos, habilidades y valores relevantes. En este contexto, debemos considerar sus experiencias de aprendizaje, los recursos utilizados en este proceso y las formas en que este aprendizaje puede integrarse en la formación profesional y la práctica clínica.

Cada sesión duró entre 30 y 60 minutos y se llevó a cabo de manera individual. Cada entrevista continuó hasta que el entrevistador y el participante coincidieron en que habían discutido todos los aspectos del fenómeno objeto de investigación. Cada entrevista fue grabada y transcrita para su posterior análisis.

Se implementaron diversas estrategias para minimizar posibles sesgos en la recolección de datos y fortalecer la credibilidad y transparencia del proceso. En primer lugar, se solicitó la opinión de colegas y expertos externos para identificar interpretaciones sesgadas que el investigador principal pudiera haber pasado por alto. Además, se mantuvo un diario de investigación reflexivo para registrar pensamientos, suposiciones y decisiones analíticas, fomentando así la autorreflexión y la conciencia de la influencia del investigador en el estudio. Por último, el investigador reconoció explícitamente su posición y cualquier influencia potencial derivada de su trayectoria profesional y personal. También comunicó abiertamente estos aspectos a los participantes para mejorar la integridad y la fiabilidad del estudio.

Las grabaciones se transcribieron textualmente y se verificaron para garantizar la fidelidad de los datos. A continuación, los archivos de audio se eliminaron de forma permanente una vez completadas la transcripción y la validación. Las transcripciones anonimizadas y los formularios de consentimiento informado se almacenaron en un servidor institucional protegido con contraseña, accesible únicamente para los miembros autorizados del equipo de investigación. De conformidad con las políticas institucionales y las recomendaciones internacionales sobre integridad de la investigación y protección de datos personales, los datos se conservarán durante un período de cinco años.

### Análisis y Procesamiento de Datos

El proceso de análisis de datos incluyó las siete etapas del análisis fenomenográfico descritas por Dahlgren y Fallsberg^(19,20)^: familiarización con los datos, condensación, comparación, agrupación, articulación, etiquetado y contraste. Inicialmente, este proceso se realizó de forma manual, aunque posteriormente se utilizó el software Atlas.Ti versión 9 para apoyar este análisis. Para garantizar el rigor de esta investigación, se utilizaron múltiples medidas, entre ellas la identificación de las ideas preconcebidas de los investigadores sobre el fenómeno objeto de estudio, la garantía de una expresión genuina del fenómeno con la ayuda de facilitadores, el logro de un consenso mediante la negociación y el uso de los siguientes criterios principales, descritos por Marton y Booth, para juzgar la calidad de las categorías identificadas^(20)^: 1) cada categoría revela un aspecto diferente de la manera en que se experimenta el fenómeno; 2) las categorías se caracterizan por relaciones lógicas y claras entre sí; y 3) los resultados finales son parsimoniosos (es decir, se emplean el menor número posible de categorías).

Se utilizaron múltiples medidas para garantizar el rigor y la calidad de la investigación. La naturaleza iterativa del estudio permitió mantener la credibilidad, la consistencia, la transferibilidad y la fiabilidad^(20)^. En primer lugar, la credibilidad se basó en la demostración de similitudes y diferencias respaldadas por datos de transcripciones, utilizando extractos de las entrevistas para fundamentar las categorías. En segundo lugar, la consistencia se estableció manteniendo categorías separadas y exclusivas y estableciendo la correspondencia entre los resultados y los estudios relacionados. En tercer lugar, la transferibilidad y la fiabilidad se desarrollaron mediante el uso de procedimientos metodológicos adecuados para obtener datos consistentes y de alta calidad para el análisis. Esto implicó un diseño meticuloso de las preguntas de la entrevista y la documentación de cada etapa del proceso de investigación.

### Aspectos Éticos

El estudio fue aprobado por el Comité de Ética en Investigación de la Facultad de Medicina de la *Universidad del Desarrollo* (n.º 2019-091) y por el Comité de Bioética de la *Universidad de Barcelona* (IRB00003099). El estudio se ajustó a los principios de voluntariedad, confidencialidad y anonimato de los participantes, tal como se refleja en la firma de un consentimiento informado.

## RESULTADOS

Los resultados del análisis de las diferencias en las percepciones de los agentes clave se han clasificado en dos categorías:

### A – Enfoque Teórico-Experiencial

Las autoridades académicas identificaron la alfabetización intercultural como un pilar fundamental en la formación de los profesionales de la salud.

*Lo veo como una alfabetización intercultural ...... Estoy seguro de que habrá formas de abordarlo eventualmente —no solo en un año, sino en varios años— como un tema importante.* (I1 AUT)

Las autoridades académicas también hicieron hincapié en que el enfoque teórico inicial debe ser continuo y no limitarse a una sola asignatura, sino integrarse a lo largo de toda la formación académica, abordando así este componente de manera sistemática y progresiva.

*Creo que lo haría de manera progresiva en el plan de estudios, primero creando conciencia de manera teórica en los primeros años y luego insertándolo de alguna manera.* (I9 AUT)

Desde la perspectiva de las autoridades académicas, existen diversas estrategias de enseñanza-aprendizaje. Estas estrategias incluían tanto clases magistrales como investigación aplicada. Además, se pueden utilizar estrategias metodológicas basadas en novelas para enseñar medicina narrativa.

... *en cuanto a las novelas, se me ocurre... en las que uno vive la experiencia del protagonista en esa cultura, incluyendo lo que sucede. Esa podría ser una forma interesante de enseñar a los estudiantes*. (I4 AUT)

Entre la variedad de estrategias de enseñanza-aprendizaje destacadas por las autoridades académicas, los portafolios fueron un ejemplo notable.

*Hacerlo una competencia en la que se trabaje desde el primer día en que ingresan, pidiendo a los propios estudiantes que identifiquen su propia cultura, su familia, sus antepasados, incluso preguntando, no sé, de dónde viene tu nombre, haciendo ejercicios diarios, exponiéndolos a casos, situaciones, periódicos, enfocándose cada día en algo relacionado con la interculturalidad*. (I3 AUT)

Por su parte, los docentes indicaron que la interculturalidad en la formación de los profesionales de la salud se reconoce principalmente como un componente implícito del plan de estudios, a través de diversas actividades de enseñanzaaprendizaje. En este contexto, identificaron la enfermería como el único programa que incorpora explícitamente este tema en los contenidos de clase. A diferencia de las autoridades académicas, los docentes reconocieron la relevancia de un enfoque teórico-experiencial en su enseñanza; sin embargo, expresaron opiniones divergentes respecto a su implementación y al grado de énfasis que debería recibir dentro del plan de estudios.

Aunque tanto los profesores como las autoridades académicas valoraron la transmisión de contenidos teóricos relacionados con los conceptos fundamentales de la interculturalidad, los profesores hicieron hincapié en la importancia de la aplicación práctica de dichos contenidos. Afirmaron que estrategias como los talleres, el trabajo en grupo, los debates y las simulaciones con pacientes estandarizados son esenciales para reforzar el aprendizaje y cultivar las CCI de manera efectiva.

*Siempre hay que empezar con conceptos, ideas, libros y artículos; siempre hay que comenzar con algo teórico, pero ese componente teórico se complementa con elementos o herramientas prácticas, como los talleres —talleres como los que dirige Andrés, que creo que son extremadamente necesarios*. (I12 TEA-NUR)

*La participación, o el enfoque, el trabajo en equipo y también los juegos de rol. El uso de imágenes, trabajar con personas de diferentes culturas. Debe ser algo que deje una impresión duradera, como cuando vas a una clase y entra un paciente, o cuando invitas a la familia del paciente; los estudiantes nunca lo olvidan.* (I14 TEA-ST)

*Desarrollar escenarios de simulación intercultural y, tal vez, incorporar escenarios de alta fidelidad en los que el estudiante deba interactuar con un usuario, una persona —por ejemplo, alguien que no hable español—; estoy pensando, por ejemplo, en una persona mapuche o haitiana— y entrar en un escenario de simulación en el que deba aplicar lo que ha aprendido.* (I12 TEA-NUR)

Otra estrategia de enseñanza que los profesores de medicina y nutrición consideraron fundamental se refiere al aula multicultural, que implica la participación de estudiantes y profesores de diferentes culturas (por ejemplo, de diferentes países o con diferentes géneros, etnias o edades), lo que puede promover el uso de estrategias de enseñanza como la discusión y el debate, junto con la exposición a la diversidad cultural en términos de opiniones y perspectivas.

*Creo que también sería maravilloso poder aprender de profesores de otras culturas y junto a estudiantes de diferentes culturas.* (I16 TEA-MED)

Los profesores de enfermería y fisioterapia tendían a centrarse más en la aplicación directa de este conocimiento a situaciones de enseñanza experiencial, y destacaron la importancia de casos clínicos específicos con respecto a un aprendizaje efectivo y significativo.

*Por lo general, trabajamos mucho con casos clínicos en los que nos ponemos en la situación de atender a un paciente, y ellos aplican el enfoque intercultural al caso en cuanto a cómo lo abordarían y qué preguntas harían*. (I13 TEA-NUR)

*La incorporación de casos clínicos. Trabajamos mucho en eso*. (I15 TEA-PHY)

En cuanto a las estrategias docentes, los profesores de enfermería también destacaron la incorporación de facilitadores culturales. Estas personas, también conocidas como traductores, facilitadores lingüísticos y/o mediadores culturales, se dedican a facilitar la comunicación entre el equipo de salud y la población migrante o las poblaciones nativas. Participan en actividades clínicas en diferentes centros de salud y son invitados a participar en actividades teóricas con los estudiantes.

*También creo que podría ser importante abordar el papel del facilitador intercultural como miembro integral del equipo de salud; creo que esto sería muy relevante.* (I13 TEA-NUR)

De manera similar, los miembros del cuerpo docente enfatizaron que la interculturalidad debe entenderse como un componente integral de la profesionalidad en la atención de la salud, y que su enseñanza debe estar alineada con los valores éticos y profesionales fundamentales. En este sentido, es esencial abordar y corregir los prejuicios a través de actividades como talleres de sensibilización cultural o visitas a una *ruca* con una *machi* mapuche (una intermediaria entre mundos). Estas personas están conectadas con el reino sobrenatural y sus espíritus protectores, que les otorgan poderes para combatir a los espíritus malévolos; salvaguardan el bienestar de sus pacientes y de la comunidad, actuando como sanadoras, particularmente en el caso de las mujeres. Del mismo modo, los estudiantes pueden participar en ejercicios de juego de roles que simulen situaciones interculturales.

*Sí, eliminar los prejuicios, creo. Porque estaba pensando en nuestra primera visita a una ruca mapuche... la clase estaba compuesta casi en su totalidad por mujeres. La machi salió y pidió que los hombres de la clase entraran primero, y luego las mujeres. Como la cultura mapuche es patriarcal, a partir de ese momento las estudiantes se molestaron; no les pareció apropiado... Necesitamos eliminar el prejuicio de que mi forma de pensar es la única válida.* (I12 TEA-NUR)

Desde la perspectiva de los estudiantes, se identificaron otras estrategias de enseñanza-aprendizaje que difieren de las destacadas por otros agentes clave, como los informantes culturales clave, por ejemplo, un miembro de una población indígena (mapuche) o una persona de cierta identidad de género (transgénero) que participa en talleres junto a los estudiantes.

*Poder traer a personas que nos den testimonio es una herramienta clave para conocer a diferentes grupos de personas. Además, las experiencias de nuestros profesores son herramientas y factores clave que pueden ayudarnos enormemente a comprender y aprender sobre este tema*. (I22 NUR-STU)

Los estudiantes también mencionaron cursos o actividades basados en el enfoque de enseñanza del aprendizaje colaborativo internacional en línea (collaborative online international learning – COIL), que representa una estrategia innovadora para enseñar la interculturalidad a los estudiantes de salud.

*O, por ejemplo, compartir ciertos cursos con personas de otras universidades, de otros países, como suelen hacer algunas plataformas de cursos*. (I19 MT-STU)

### B – Experiencia Directa en la Práctica Clínica

En este estudio, las autoridades académicas hicieron hincapié en la relevancia de la integración de la experiencia práctica en el proceso de enseñanza de la interculturalidad. La integración de la teoría y la práctica no debe tratarse de forma aislada, sino que debe incluirse de manera cohesiva y continua en el plan de estudios y considerarse un componente esencial de una estrategia educativa. En particular, se destacó la importancia de las rotaciones clínicas en comunidades culturalmente diversas a nivel de atención primaria y hospitalaria, en cuyo contexto los estudiantes interactúan directamente con pacientes de diferentes culturas en situaciones reales. Esta inmersión práctica ayuda a los estudiantes a desarrollar habilidades como la empatía y la adaptabilidad, asegurando así que los futuros profesionales sean capaces de aplicar los conocimientos interculturales en su práctica diaria.

*Al final, todo se aprende a través de la práctica: escuchando y observando. Puedes leer millones de artículos y libros, pero nunca será lo mismo que lo que experimentas, como cuando vas a un entorno clínico donde se atiende a personas de diferentes culturas; esa es una forma poderosa de aprendizaje*. (I6 AUT)

*Creo que los estudiantes deben estar expuestos a diferentes situaciones clínicas experienciales —con pacientes, con personas— en diferentes entornos*. (I3 AUT)

Las autoridades académicas también identificaron el modelado por parte de los tutores clínicos como una estrategia de enseñanza fundamental en el proceso de enseñar interculturalidad a los estudiantes de salud. La interacción entre tutores y estudiantes en un contexto clínico permite a los futuros profesionales observar y aprender a aplicar las habilidades interculturales en situaciones reales, y los tutores sirven como ejemplos experienciales en este contexto.

*Creo que los conceptos se pueden enseñar en el aula durante los primeros años. Sin embargo, más adelante deben desarrollarse a través del modelado. En ese momento, el tutor debe estar inmerso en este proceso para poder transmitirlo eficazmente a los estudiantes*. (I10 AUT)

... *el modelado; los estudiantes no hacen lo que tú dices, los estudiantes hacen lo que tú haces. No sé; en mi caso, por ejemplo, nunca he olvidado a aquellos profesores que me enseñaron bien, y nunca he olvidado a aquellos profesores que me enseñaron mal*. (I2 AUT)

Tanto los profesores como las autoridades académicas consideraban que la experiencia directa en el ámbito clínico era esencial para el proceso de aprendizaje de la interculturalidad. Sin embargo, los enfoques que mencionaron variaban en cuanto a cómo debían ser esas experiencias. Hacen hincapié en la importancia de preparar adecuadamente a los estudiantes antes de su exposición a diversos entornos clínicos, así como de acompañarlos durante estas experiencias con el objetivo de garantizar que puedan adquirir las competencias necesarias.

*Intentaría asegurarme de que todos mis estudiantes tuvieran una experiencia con un paciente de otro país, con un sistema de creencias diferente, o de una etnia o raza diferente. Por ejemplo, trataría de asegurarme de que no sientan ese miedo cuando se enfrenten a alguien de otra cultura en el futuro.* (I16 TEA-MED)

Una estrategia de enseñanza que destacaron los profesores de enfermería se refería a la clínica para migrantes, que es una actividad práctica clave que puede facilitar el desarrollo de las CI entre los estudiantes de salud. Estas experiencias prácticas brindan una oportunidad única para que los estudiantes se enfrenten a la realidad de atender a poblaciones vulnerables y culturalmente diversas.

*Creo que una de estas formas es a través de la clínica de migrantes, donde el estudiante tiene la oportunidad de interactuar de manera muy positiva con pacientes que recién ingresan al sistema de salud.* (I13 TEA-NUR)

Los profesores enfatizaron el hecho de que la evaluación de la interculturalidad no debe limitarse a exámenes teóricos, sino que también debe incluir herramientas que ayuden a medir el desempeño práctico de los estudiantes en entornos reales. Los profesores mencionaron la importancia de evaluar la capacidad de los estudiantes para adaptarse a situaciones interculturales, así como su competencia para interactuar con pacientes de diversos orígenes culturales.

*Creo que en cursos más avanzados, por ejemplo, en el tercer, cuarto y quinto año, los criterios de evaluación — especialmente durante las rotaciones clínicas— deberían incluir una forma explícita de evaluar si el estudiante es capaz de integrar la interculturalidad en su práctica de la salud*. (I12 TEA-NUR)

Desde la perspectiva de los estudiantes, además de las estrategias mencionadas por las autoridades académicas y los miembros del cuerpo docente, se identificaron experiencias prácticas no obligatorias, como programas de voluntariado tanto a nivel local como nacional. Estas oportunidades permiten la interacción directa con poblaciones diversas, lo que facilita el desarrollo de competencias interculturales en contextos del mundo real.

... *Se me ocurren ejemplos de esto en programas como Operation Smile, Calle Salud e iniciativas similares...*(I17 MED-STU)

... *la universidad también nos da la oportunidad de participar en trabajo voluntario, y creo que es una de las mejores formas de conocer las diferentes culturas que existen no solo a nivel de la ciudad, como en Santiago, sino también a nivel nacional*. (I23NUR-STU)

Los estudiantes también mencionaron los programas de intercambio académico internacional, que identificaron como una estrategia pedagógica altamente eficaz que puede utilizarse para cultivar las CI entre los estudiantes de salud.

*Los intercambios, que van y vienen*. (I18 MED-STU)

... *hay diferentes intercambios para diversos lugares, y también están muy abiertos a la posibilidad de que lleguen aquí estudiantes de intercambio*. (I22 NUR-STU)

En resumen, se identifican dos enfoques: (A) Enfoque teórico-experiencial: los expertos y el cuerpo docente enfatizan la importancia de combinar la teoría con estrategias experienciales, tales como talleres, simulaciones y aprendizaje colaborativo internacional; (B) Experiencia directa en la práctica clínica: se hace hincapié en la inmersión en contextos del mundo real a través de rotaciones clínicas, tutoría y participación en clínicas para migrantes. Los estudiantes destacan el valor del voluntariado y el intercambio académico.

## DISCUSIÓN

Nuestro estudio presenta varias limitaciones, que se refieren principalmente a posibles fuentes de sesgo y a la generalizabilidad limitada de estos hallazgos. Estas limitaciones incluyen un posible sesgo de recuerdo o respuestas subjetivas. Además, el diseño de esta investigación, que implicó una metodología fenomenográfica, se centra en las variaciones entre las experiencias más que en la generalización.

Los procesos de enseñanza y aprendizaje de la interculturalidad en el contexto de la formación de los profesionales de la salud se caracterizan por diferencias notables en las percepciones y los enfoques que destacan las autoridades académicas, los profesores y los estudiantes. Esta investigación reveló que la formación en interculturalidad entre los profesionales de la atención de salud es un proceso complejo, que debe tener en cuenta tanto la adquisición de conocimientos teóricos como la aplicación práctica de habilidades interculturales en la educación sanitaria, permitiendo así que los futuros profesionales de la atención de salud se sientan preparados para brindar atención o que puedan centrarse en los pacientes en un contexto intercultural, ya que estudios previos han sugerido que persiste una falta de preparación entre los profesionales de la atención de salud^(21,22)^.

Los hallazgos de este estudio en relación con las autoridades académicas subrayan la necesidad de una alfabetización intercultural que sea continua y transversal. Este enfoque en la alfabetización debe integrarse en todo el plan de estudios académico, lo que representa un desafío significativo para las estructuras institucionales en sus esfuerzos por incorporar este contenido en los planes de estudio de todos los programas de salud. Este enfoque permite a las partes interesadas relevantes garantizar que los estudiantes interioricen estos principios desde el inicio de su formación. Este enfoque concuerda con investigaciones que han destacado la importancia de incorporar de manera explícita y sistemática la interculturalidad en el plan de estudios desde el inicio del proceso de formación^(4)^. Este enfoque implica no solo la inclusión de contenidos teóricos relacionados con la diversidad cultural, sino también la promoción de la reflexión crítica sobre los propios prejuicios y sesgos^(12)^. Esta reflexión puede facilitarse mediante un análisis de las experiencias vividas, lo que ayuda a los estudiantes a organizar y comunicar sus pensamientos y les permite determinar si realmente comprenden un tema determinado^(23,24)^. En este sentido, los profesores creen que este enfoque debe estar respaldado por diversas estrategias pedagógicas, como los estudios de casos y los portafolios reflexivos. Se ha informado que estas estrategias sensibilizan eficazmente a los estudiantes sobre la importancia de una atención médica culturalmente competente.

En cuanto al enfoque teórico-experiencial, los docentes de este estudio hicieron hincapié en que la interculturalidad no puede examinarse únicamente desde un punto de vista teórico. Más bien, debe ir acompañada de estrategias de enseñanza y aprendizaje experienciales que permitan a los estudiantes aplicar sus conocimientos a situaciones de la vida real en el aula. Esto fomenta la introspección, el intercambio de experiencias, el aprendizaje colaborativo y la conciencia de la diversidad cultural^(12,13,22)^.

Además de las estrategias tradicionales identificadas por este estudio, como el aprendizaje basado en problemas, el análisis de casos y las simulaciones con pacientes estandarizados, la inclusión de pacientes de diferentes géneros o con diferentes creencias puede proporcionar un entorno controlado en el que los estudiantes puedan mejorar sus habilidades y su confianza. Otras posibilidades se refieren a la participación en talleres con informantes culturales y al contacto directo con poblaciones diversas. Por ejemplo, la interacción con pueblos indígenas, como los mapuches, o con personas de diversas identidades de género, puede brindar a los estudiantes una comprensión directa de las barreras que estas poblaciones enfrentan en el sistema de atención de salud y de sus necesidades^(8,12,22)^. La literatura ha informado que este tipo de intervención puede facilitar el desarrollo de habilidades esenciales relacionadas con la atención de poblaciones diversas y promover una mayor sensibilidad hacia los determinantes socioculturales de la salud^(3,13)^. Además, las investigaciones sobre el contacto intergrupal han sugerido que la exposición a grupos que históricamente han enfrentado prejuicios puede transformar las actitudes negativas, promoviendo así una perspectiva más comprensiva y empática entre los futuros profesionales de la salud^(23)^.

Los docentes incluidos en este estudio también indicaron que las aulas y los profesores interculturales pueden utilizarse en este contexto. El contacto intercultural entre profesores de diferentes orígenes y los estudiantes ofrece valiosas oportunidades para que estos desarrollen la CI^(10)^. En primer lugar, la diversidad de opiniones y perspectivas puede destacarse en los debates grupales, que pueden enriquecerse gracias a la heterogeneidad del grupo. En segundo lugar, los estudiantes observan cómo los comportamientos culturales que han aprendido previamente de manera teórica pueden aplicarse a situaciones cotidianas dentro del aula. En tercer lugar, la interacción en aulas multiculturales permite a los estudiantes adquirir conocimientos sobre diferentes culturas directamente de sus profesores y compañeros, enriqueciendo así su comprensión^(13)^.

Otra nueva metodología emergente identificada por los estudiantes es el enfoque COIL, que facilita la interacción entre estudiantes de diferentes países y culturas, enriqueciendo así el aprendizaje intercultural sin necesidad de movilidad física^(24)^. Según estudios recientes, la implementación del enfoque COIL en el contexto de la educación para la salud es una herramienta eficaz que puede utilizarse para aumentar la CI^(25)^.

La experiencia directa en la práctica clínica es también un aspecto fundamental de la educación intercultural^(22)^, y nuestros datos respaldan el vínculo entre las experiencias clínicas y la preparación para la atención intercultural. La interacción directa con pacientes migrantes, personas de diferentes géneros y miembros de otros grupos culturales, lo que a menudo conlleva dificultades en términos de factores lingüísticos, sociales y culturales, permite a los estudiantes aplicar de manera efectiva sus conocimientos teóricos sobre la interculturalidad. En este contexto, una estrategia de enseñanza-aprendizaje implica rotaciones o prácticas clínicas en comunidades diversas. Esto brinda a los estudiantes oportunidades invaluables para aplicar sus conocimientos en situaciones reales, un enfoque que ha recibido un fuerte respaldo de la literatura^(22,26)^.

El ejemplo de los docentes clínicos (tutores) es fundamental para la enseñanza de la interculturalidad en el contexto de la formación de los profesionales de la salud. Estos tutores no solo sirven de referencia en cuanto a la aplicación de la CI en la práctica clínica diaria, sino que también refuerzan el aprendizaje teórico de los estudiantes, fortaleciendo así su confianza en su capacidad para manejar entornos clínicos culturalmente diversos. La literatura ha respaldado la afirmación de que un modelado eficaz contribuye significativamente al desarrollo de la empatía y la capacidad de comunicación intercultural entre los estudiantes, aumentando así su preparación para actuar en situaciones clínicas que involucran a pacientes de diferentes contextos culturales^(6,27)^. Sin embargo, la literatura también ha destacado múltiples barreras que pueden obstaculizar la integración de esta formación, incluyendo restricciones institucionales, resistencia al cambio por parte de los estudiantes y una falta de apoyo continuo a las iniciativas educativas en este contexto^(28)^.

Otra metodología mencionada en este contexto fue la clínica para migrantes. Esta ofrece un entorno de aprendizaje rico y estimulante y promueve el desarrollo de habilidades fundamentales, como la empatía, la resolución de problemas y la adaptación cultural. Estas habilidades son clave para garantizar que los futuros profesionales de la salud brinden una atención inclusiva y culturalmente competente^(10,22)^.

Los estudiantes destacaron la exposición directa a contextos interculturales a través de actividades como los intercambios o el voluntariado, que han demostrado ser facilitadores clave para el desarrollo de estas competencias^(29,30)^. Estas experiencias permiten a los estudiantes interactuar directamente con poblaciones culturalmente diversas tanto a nivel local como internacional, fomentando así una mayor apertura y sensibilidad cultural, que son elementos esenciales de una atención sanitaria de calidad en un mundo cada vez más diverso. El aprendizaje a través del voluntariado expone a los estudiantes a diferentes contextos culturales, mejorando así no solo su comprensión de las particularidades de cada grupo, sino también su capacidad para adaptar sus prácticas a las necesidades culturales específicas de las personas a las que atienden^(29)^. La participación en estos programas es una herramienta pedagógica eficaz que debería integrarse más ampliamente en los planes de estudios de educación en salud^(30)^.

Por último, nuestros resultados subrayan la importancia de evaluar la CI, particularmente en entornos clínicos. Según la literatura, la evaluación de la interculturalidad debería ir más allá de los exámenes teóricos para incluir observaciones en entornos clínicos, simulaciones con retroalimentación formativa y análisis de portafolios reflexivos^(10,12,22)^.

La evaluación de la competencia cultural en los planes de estudios de formación en salud es un área que puede desarrollarse aún más. Diseñar un sistema de evaluación sólido y estructurado que incorpore rúbricas claras y métodos de evaluación tanto formativos como sumativos es esencial para garantizar un aprendizaje significativo.

Los futuros investigadores podrían replicar este estudio en otras universidades y explorar más a fondo cómo se enseña y se aprende la interculturalidad en los programas de salud, ya que la investigación sobre este tema sigue siendo limitada.

## CONCLUSIÓN

Este estudio destaca la relevancia de la integración de la interculturalidad en la formación de manera transversal. Es necesario un enfoque continuo que combine la teoría con la práctica a lo largo del proceso educativo. El proceso de enseñanza de la interculturalidad debe basarse en una combinación de experiencias, metodologías colaborativas de enseñanza-aprendizaje y herramientas de reflexión crítica que puedan ofrecer a los estudiantes una comprensión más profunda de la diversidad cultural.

El ejemplo de los tutores clínicos y la experiencia práctica en entornos culturalmente diversos, como las clínicas para migrantes, son componentes esenciales. Estas experiencias no solo facilitan la adquisición de habilidades técnicas, sino que también promueven el desarrollo de la empatía y la adaptabilidad, que son elementos fundamentales de una formación inclusiva y culturalmente sensible.

Esta investigación aporta una contribución novedosa al campo al ofrecer un análisis fenomenográfico exhaustivo de cómo se enseña y se aprende la interculturalidad en la educación en salud. A diferencia de estudios anteriores, que se han centrado principalmente en evaluar la competencia cultural a través de intervenciones aisladas o enfoques teóricos, este estudio integra las perspectivas de autoridades académicas, profesores y estudiantes para revelar la complejidad y variabilidad de estas experiencias. La identificación de estrategias emergentes —como la inclusión de COIL, las aulas interculturales y los modelos clínicos experienciales— aporta nuevas perspectivas sobre cómo se puede desarrollar eficazmente la CI. Estos hallazgos amplían el conocimiento actual al enfatizar que la educación intercultural debe abordarse como un proceso continuo y multidimensional que une la teoría, la práctica y la reflexión a lo largo de la formación profesional.

## Data Availability

Todo el conjunto de datos que apoya los resultados de este estudio fue publicado en el propio artículo.
